# Comparison of spherical equivalent and estimated prevalence of myopia in school-age children between Shanghai and Yunnan in China

**DOI:** 10.3389/fmed.2025.1571470

**Published:** 2025-05-14

**Authors:** Tao Li, Xiandong Liu, Xiaodong Zhou

**Affiliations:** ^1^Department of Ophthalmology, Jinshan Hospital, Fudan University, Shanghai, China; ^2^Department of Ophthalmology, Zhenyuan People’s Hospital, Yunnan, China; ^3^Jinshan District Eye Disease Prevention and Treatment Center, Shanghai, China

**Keywords:** spherical equivalent, myopia prevalence, Han ethnicity, ethnic minority, children

## Abstract

**Purpose:**

The purpose of this study was to compare spherical equivalent (SE) and estimated prevalence of myopia in school-age children between Shanghai and Yunnan in China, and to investigate the differences in SE and estimated prevalence of myopia between Han ethnicity and ethnic minorities in Yunnan.

**Methods:**

This was a retrospective observational study, which enrolled one primary school and one junior high school in Shanghai, and two primary schools and two junior high schools in Yunnan in 2021. Refraction was measured using autorefractors without cycloplegia. Pearson correlation analysis, Chi-square test and multiple linear regression (MLR) were used for analysis.

**Results:**

A total of 2,744 children in Shanghai and 1,769 children in Yunnan were examined, respectively. Less hyperopic SE was observed in Grades 1 and 2 in Shanghai compared to Yunnan (both *P* < 0.001). No significant difference in median SE between Han ethnicity and ethnic minorities in Yunnan was found in each grade (all *P* > 0.05). The estimated prevalence of myopia in Grades 1 (13.3% vs. 2.6%, *P* < 0.001) and 2 (16.1% vs. 7.6%, *P* = 0.003) were higher in Shanghai than in Yunnan, whereas the estimated prevalence of myopia of in Grade 7 (52.0% vs. 68.1%, *P* = 0.042) was lower in Han ethnicity than in ethnic minorities in Yunnan. MLR showed that the coefficient for age was −0.3351 (*p* < 0.001).

**Conclusion:**

Spherical equivalent and estimated prevalence of myopia of school-age children in Yunnan are similar to Shanghai. Furthermore, a similar situation exists between Han ethnicity and ethnic minorities.

## 1 Introduction

Myopia is becoming a worldwide public health concern with rapidly increasing prevalence (1, 2). Approximately 50% of the global population (4,758 million people) is projected to suffer from myopia in 2050 (3). The prevalence of myopia in children is especially high in East and Southeast Asia, approximately 80%–90% at 17 and 18 years-old (3). Furthermore, the prevalence of myopia is even more than 90% at 18 years-old in Shanghai, China (4). It is reported that Chinese population have a higher myopia prevalence than non-Chinese population in multiethnic countries (5, 6). The myopia and myopia-related complications can lead to visual impairment and substantial economic burden in China (7, 8), which will interfere with children’s study and family living standards.

China is a large country with Han ethnicity (more than 91%) and 55 ethnic minorities in different regions (9). Previous studies reported that the prevalence of myopia in urban areas [e.g., Shanghai (10–12), Beijing (13, 14), Guangzhou (15, 16), etc.,] was higher than rural areas [e.g., Yunnan (17–19), Xinjiang (20, 21), etc.,] and the prevalence of myopia in Han ethnicity was higher than ethnic minorities (18–21). These differences may be attributed to variations in socioeconomic status and education levels. However, it is unclear whether the prevalence of myopia is increasing over time in rural China due to dramatic changes in environmental exposures and lifestyles, and whether the gap is narrowing among different ethnic populations.

Shanghai is located in southeast China, whereas Jinshan district is located on the southwest of Shanghai. Jinshan district has a total population of more than 820,000 people, which is mainly composed of Han ethnicity, approximately accounting for 99% of the population. Yunnan Province is located on the southwestern border of China, whereas Zhenyuan County is located on the southwest of Yunnan Province. Zhenyuan County, the youngest autonomous county in China, was born on 3 February 1990. It has a total population of more than 200,000 people, predominantly composed of Han, Yi, Hani, Lahu and other ethnic minorities. Han ethnicity accounts for 43.1% of the population, making it a minority compared to other ethnic groups. The purposes of this study were: (1) to investigate the differences in spherical equivalent (SE) and estimated prevalence of myopia between Jinshan District, Shanghai and Zhenyuan County, Yunnan Province; and (2) investigate the differences in SE and estimated prevalence of myopia between Han ethnicity and ethnic minorities in Zhenyuan County.

## 2 Materials and methods

### 2.1 Subjects

This was a retrospective observational study conducted in Jinshan District, Shanghai and in Zhenyuan County, Yunnan Province, China. The study included one primary school and one junior high school in Shanghai, and two primary schools and two junior high schools in Yunnan in 2021. The exclusion criteria were: ethnic minorities in Shanghai; systemic diseases affecting ocular refraction (e.g., Marfan syndrome); severe ocular diseases and surgeries (e.g., cataract, glaucoma); and orthokeratology lens correction. The enrolled children were divided into nine groups according to their grade.

### 2.2 Examination

The children’s refraction examinations were conducted on September 8 and 23 in Jinshan District, Shanghai, and from October 20 to 28 in Zhenyuan County, Yunnan. Similar to our previous studies (22–24), refraction was measured using autorefractors (Shanghai: RK-F1, Canon Corporation, Tokyo, Japan; Yunnan: KR-800, Topcon Corporation, Tokyo, Japan) without cycloplegia. The average value of three reliable measurements was used for analysis.

### 2.3 Statistical analysis

Both eyes of each child were examined, but only data from the right eye were used for analysis. Spherical equivalent (SE) was calculated as the sum of sphere power and half of cylinder power. According to our previous studies (22, 23), myopia was defined as SE of at least −1.00 D.

SPSS V. 17.0 software was used for data analysis. Since SE was not normally distributed according to the Kolmogorov-Smirnov test, the Kruskal-Wallis test and post hoc Mann–Whitney U tests were used for statistical comparisons. Pearson correlation analysis was used to evaluate the relationship between SE and grades. Chi-square test was used to analyze the differences in the gender and estimated prevalence of myopia between Shanghai and Yunnan, and between Han ethnicity and ethnic minorities in Yunnan.

In addition, multiple linear regression (MRL) was used to establish SE prediction model. Based on existing measurements, this study examines the effects of region (x_1_), ethnicity (x_2_), and age (x_3_) on SE (y). The regression model used in this study was:


y=β+0βx1+1βx2+2βx3+3ε

Region had two values: 1 and 2 (1 represents Shanghai Jinshan District, 2 represents Zhenyuan County, Yunnan). Ethnicity also had two values: 1 and 2 (1 represents Han Chinese, 2 represents ethnic minorities). After establishing the regression equation, significance tests were conducted on the variables. All *P*-values were two-sided and considered statistically significant when less than 0.05.

## 3 Results

The characteristics for each grade in Shanghai and Yunnan are shown in Table 1. A total of 2,744 children in Shanghai and 1,769 children in Yunnan were examined. There were 1,438 (52.4%) boys in Shanghai and 933 (52.7%) boys in Yunnan (*P* = 0.825). Furthermore, Han ethnicity accounted for 26.2% of the children in Yunnan.

**TABLE 1:** Characteristics for each grade in Shanghai and Yunnan.

Grade	Shanghai		Yunnan		
	*N*	Boys (%)	*N*	Boys (%)	Han ethnicity (%)
1	348	191 (54.9)	192	88 (45.8)	26 (13.5)
2	310	164 (52.9)	197	94 (47.7)	47 (23.9)
3	388	192 (49.5)	210	105 (50.0)	55 (26.2)
4	429	224 (52.2)	191	93 (48.7)	67 (35.1)
5	444	238 (53.6)	207	113 (54.6)	44 (21.3)
6	275	150 (54.5)	202	111 (55.0)	57 (28.2)
7	195	100 (51.3)	191	107 (56.0)	50 (26.2)
8	175	87 (49.7)	189	112 (59.3)	52 (27.5)
9	180	92 (51.1)	190	110 (57.9)	66 (34.7)

### 3.1 Spherical equivalent

**FIGURE 1:**
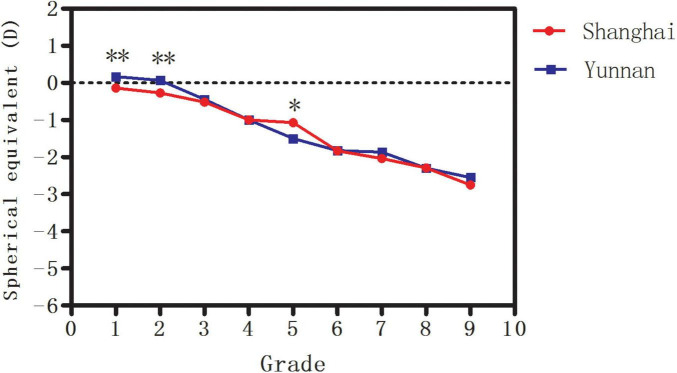
The median spherical equivalent (SE) in Shanghai and Yunnan. *Significant at the 0.05 level; **Significant at the 0.001 level.

The median, interquartile range (IQR), and range of SE for the children of each grade in Shanghai are shown in Table 2. The median SE decreased from −0.13 D in Grade 1 to −2.25 D in Grade 9 (*P* < 0.001). The median, IQR and range of SE for the children of each grade in Yunnan were shown in Table 3. The median SE decreased from +0.25 D in Grade 1 to −2.38 D in Grade 9 (*P* < 0.001). Less hyperopic SE was observed in Grades 1 and 2 in Shanghai compared to Yunnan (both *P* < 0.001; Figure 1). However, less myopic SE in Grade 5 was observed in Shanghai (*P* = 0.001; Figure 1) than in Yunnan. All these differences were too small to be of clinical significance.

**TABLE 2:** Spherical equivalent in Shanghai.

Grade	Median	IQR	Minimum	Maximum
1	−0.13	0.75	−2.75	+3.63
2	−0.19	0.88	−5.63	+7.00
3	−0.25	0.84	−9.88	+5.75
4	−0.50	1.63	−13.25	+2.75
5	−0.63	1.75	−8.00	+4.13
6	−1.50	2.25	−9.00	+5.25
7	−1.50	2.25	−8.50	+0.75
8	−1.75	2.75	−9.00	+1.50
9	−2.25	2.50	−10.25	+3.50

IQR, interquartile range.

**TABLE 3:** Spherical equivalent in Yunnan.

Grade	Median	IQR	Minimum	Maximum
1	+0.25	0.50	−8.75	+3.25
2	+0.13	0.75	−2.75	+2.13
3	−0.25	0.88	−6.00	+4.88
4	−0.75	1.75	−5.75	+3.50
5	−1.25	2.13	−9.63	+2.88
6	−1.44	2.16	−7.25	+2.63
7	−1.50	2.75	−7.63	+2.50
8	−2.13	2.63	−8.25	+2.25
9	−2.38	2.75	−8.75	+6.38

IQR, interquartile range.

**FIGURE 2:**
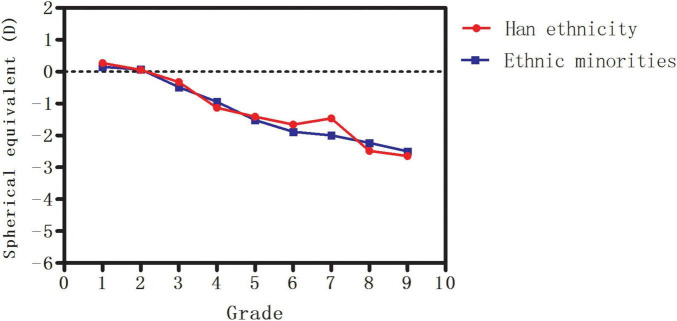
The median spherical equivalent (SE) in Han ethnicity and ethnic minorities in Yunnan.

In addition, the median SE decreased from +0.27 D in Grade 1 to −2.64 D in Grade 9 (*P* < 0.001) in Han ethnicity and from +0.15 D in Grade 1 to −2.50 D in Grade 9 (*P* < 0.001) in ethnic minorities in Yunnan. No significant difference in median SE was found in any grade between Han ethnicity and ethnic minorities (all *P* > 0.05, Figure 2).

As shown in Table 4, SE in Shanghai (R = −0.468, *P* < 0.001), SE in Yunnan (R = −0.586, *P* < 0.001), SE of Han ethnicity in Yunnan (R = −0.580, *P* < 0.001) and SE of ethnic minorities in Yunnan (R = −0.593, *P* < 0.001) were significantly correlated with grades.

**TABLE 4:** The correlations of spherical equivalent (SE) with grades.

SE	Grades	
	**R**	***P***
SE in Shanghai	−0.468	< 0.001
SE in Yunnan	−0.586	< 0.001
SE of Han ethnicity in Yunan	−0.580	< 0.001
SE of ethnic minorities in Yunan	−0.593	< 0.001

### 3.2 Estimated prevalence of myopia

**FIGURE 3:**
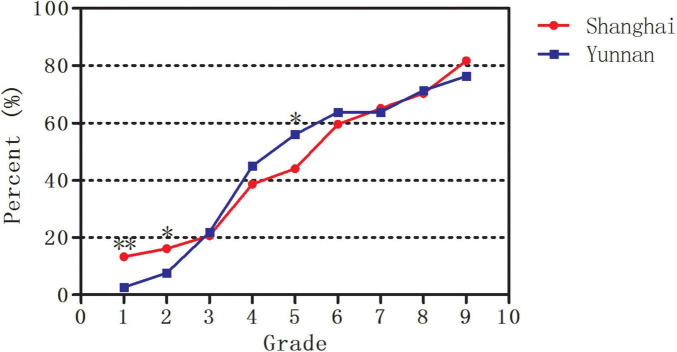
The estimated prevalence of myopia in Shanghai and Yunnan. *Significant at the 0.05 level; **Significant at the 0.001 level.

As illustrated in Figure 3, the estimated prevalence of myopia increased from 13.3% in Grade 1 to 81.7% in Grade 9 in Shanghai (*P* < 0.001) and from 2.6% to 76.3% in Yunnan (*P* < 0.001). Only the estimated prevalence of myopia in Grades 1 (13.3% vs. 2.6%, *P* < 0.001) and 2 (16.1% vs. 7.6%, *P* = 0.003) was higher in Shanghai than in Yunnan. However, the estimated prevalence of myopia in Grade 5 (44.1% vs. 56.0%, *P* = 0.003) was lower in Shanghai than in Yunnan.

**FIGURE 4:**
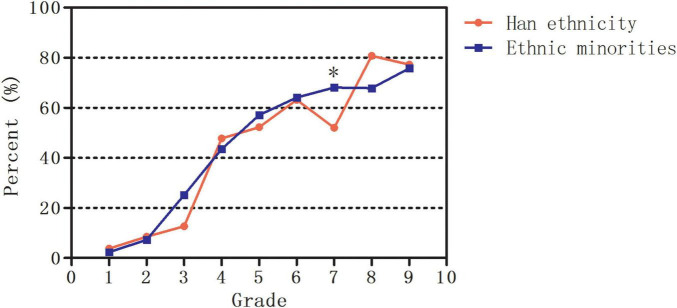
The estimated prevalence of myopia in Han ethnicity and ethnic minorities in Yunnan. *Significant at the 0.05 level.

In addition, the estimated prevalence of myopia increased from 3.8% in Grade 1 to 77.3% in Grade 9 in Han ethnicity (*P* < 0.001) and from 2.4% to 75.8% in ethnic minorities (*P* < 0.001) in Yunnan. Only the estimated prevalence of myopia in Grade 7 (52.0% vs. 68.1%, *P* = 0.042) was lower in Han ethnicity than in ethnic minorities. No significant differences in the estimated prevalence of myopia between Han ethnicity and ethnic minorities were found in other grades (all *P* > 0.05, Figure 4).

### 3.3 Spherical equivalent prediction model

The final regression equation was:


$$y\;=\;2.1353\;+\;0.1325\;x_{1}\;-\;0.0330\;x_{2}\;-\;0.3351\;x_{3}$$

Table 5 showed standard error analysis and regression coefficient significance tests for the three independent variables. The coefficient for x_1_ was 0.1325 (*p* = 0.092), implying a marginally significant positive effect of region on SE. The coefficient for x_2_ was −0.0330 (*p* = 0.695), indicating no significant ethnic difference in SE between Han ethnicity and ethnic minorities. The coefficient for x_3_ was −0.3351 (*p* < 0.001), demonstrating a significant negative effect of age on SE. With the increasing age, SE tended to be more myopic.

**TABLE 5:** Standard error analysis and regression coefficient significance tests for the three independent variables.

Variable	DF	Estimate	SE	t	*P*
Intercept	1	2.1353	0.114	18.652	< 0.001
x1	1	0.1325	0.079	1.684	0.092
x2	1	−0.0330	0.084	−0.393	0.695
x3	1	−0.3351	0.009	−35.750	< 0.001

## 4 Discussion

The present study demonstrated that less hyperopic SE and a higher estimated prevalence of myopia in Grades 1 and 2 were observed in Jinshan District, Shanghai than in Zhenyuan County, Yunnan. The children in other grades had similar SE and estimated prevalence of myopia in Shanghai and Yunnan. This may be due to the difference in preschooling systems between Shanghai and Yunnan. Almost all children in Shanghai attend preschools such as kindergartens or childcare centers, whereas most children in Yunnan may not. Thus, children in Grades 1 and 2 in Yunnan may be under less educational “pressure” than their counterparts in Shanghai. In addition, refraction in the early school years also seems to be affected by parental myopia status (25), with more myopic parents leading to more myopic refraction of children, whereas the prevalence of parental myopia may be higher in Shanghai than in Yunnan. Furthermore, the children had similar SE and estimated prevalence of myopia from Grade 1 to Grade 9 between Han ethnicity and ethnic minorities in Zhenyuan County.

The present study observed a major similar decreasing trend of SE with increasing grades in both Shanghai and Yunnan. In this study, SE had a decrease of approximately 0.24 D/year in Shanghai and 0.29 D/year in Yunnan, respectively. This age-related pattern of SE was consistent with previous studies (4, 26). Our previous study found that mean SE decreased from +0.07 ± 0.87 D to −3.10 ± 2.25 D from Grade 1 to Grade 9 in Jinshan District in 2018, with a decrease of approximately 0.35 D/year (4). Xiong et al. (26) found that SE decreased from +1.05 ± 1.17 D at 7 years-old to −2.29 ± 2.55 D at 15 years-old in Shanghai, with a decrease of approximately 0.37 D/year. However, Pan et al. (27) found that in Mojiang County, nearby Zhenyuan County, the median SEs of Grades 1 and 7 were +0.88 D and +0.15 D, respectively, with a decrease of approximately 0.12 D/year. The discrepancy may be due to the different methods of refraction measured (cycloplegia by Pan et al. (27) VS. without cycloplegia in this study), or the increasing trend of myopia in the past 5 years in Yunnan. In addition, Ding et al. divided a grade into three 4 months age blocks according to their birth month, and found that SE differences between the youngest block and oldest block in the adjacent grade can range from −0.30 D to as high as −0.80 D (28). This indicates that earlier learning may accelerate myopia progression.

In this study, the estimated prevalence of myopia in Grade 1 in Shanghai and Yunnan was 13.3% and 2.6%, respectively, and increased to 81.7% and 76.3% in Grade 9, respectively. Our previous study found that the prevalence of myopia increased from 15.7% to 87.2% from Grade 1 to Grade 9 in Jinshan District in 2018 (4), which was consistent with this study. However, the prevalence of myopia in Grades 1 and 7 in Mojiang County was 2.4% and 29.4%, respectively (27), which were similar to Grade 1 (2.6%) and lower than Grade 7 (63.9%) in Zhenyuan County in this study. Ma et al. (7) found the prevalence of myopia in children aged 5–14 years-old was higher in Shanghai than Yunnan (5–9 years-old: 18.4% vs. 4.9%; 10–14 years-old: 45.7% vs. 30.5%). However, their sample size was smaller than in this study, and no further comparisons were made for each grade. The increasing estimated prevalence of myopia in Yunnan in this study, which is gradually close to Shanghai, may be related to the continuous improvement in their education level, economic development and urbanization in the past decades. Therefore, attention should be given to the narrowing differences in environmental exposures and lifestyles. Shanghai, with a good ophthalmic public health system, can monitor the refractive development of children, and take the corresponding intervention measures. Therefore, more eye health education is also needed for children in rural areas, similar to Shanghai.

Previous studies have reported obvious genetic differences between Han ethnicity and ethnic minorities (29), which has led to the assumption that Han populations are more susceptible to myopia. Hu et al. (30) found that the prevalence of myopia in children in Grades 4 and 5 was higher in Yunnan Han ethnicity (6.48%) than Yunnan minorities (4.04%), which were lower than in Guangdong Han ethnicity (9.87%). Chin et al. (21) found that the prevalence of myopia in children aged 4–19 years-old varied significantly with ethnicity in Xinjiang: Han 27%; Hui 18%; Uyghur 13%, respectively, which demonstrated that Han children were 2.6 times and 1.8 time more likely to be myopic than Uyghur and Hui children, respectively. Compared to the Uyghur children, the Han children aged 9–13 years-old had a higher prevalence of myopia (46.9% vs. 22.0%) in Xinjiang (20). However, the children had the similar SE and prevalence of myopia from Grade 1 to Grade 9 between Han ethnicity and ethnic minorities in Zhenyuan County in this study. Furthermore, the decreasing trend of SE of Han ethnicity in Yunnan was similar to SE of ethnic minorities in Yunnan. Zhang et al. (29) found that the prevalence of myopia was 20.4% in children aged 7–12 years-old of Han ethnicity in Yunnan, which was similar to Dai ethnicity (21.0%). Qian et al. (19) found that ethnicity was not a significant associated factor for myopia in Yunnan. The genetic differences may be relatively smaller among different ethnic populations living in the same regions. IMI has reported that the differences between ethnic groups in the prevalence of myopia may be mediated by environmental exposures (31). For example, the prevalence of myopia in the population of Indian and Malay origin in Singapore is now almost as high as the Chinese, whereas the prevalence of myopia in Indians and Malays in their countries of ancestry is much lower. In addition, genetic studies have not found major differences between East Asian and European ethnic groups in the levels of myopia-associated single-nucleotide polymorphisms (SNPs) (32). Thus the similar SE and estimated prevalence of myopia in school-age children in Yunnan may be mainly due to the similar lifestyles and environmental exposures (e.g., schooling system, etc.).

In the present study, MRL showed a significant negative effect of age on SE, indicating that SE tended to become more myopic with the increase of age. MRL also showed that the coefficient of determination R^2^ was 0.222, indicating that the model could explain approximately 22.2% of the variation in SE. This suggests limitations in the current model, as factors such as region, ethnicity, and age alone cannot fully account for all factors influencing SE. Other factors such as gender, genetics, or environmental variables may play roles.

There are several limitations in this study. Firstly, a small sample size was studied. SE and estimated prevalence of myopia were analyzed in only two schools in Jinshan District, Shanghai and 4 schools in Zhenyuan County, Yunnan, which could not be representative of Shanghai and Yunnan. Secondly, SE was calculated with non-cycloplegic autorefraction, which may lead to an overestimation of myopia and estimated prevalence of myopia. Only a difference of less than −0.50 D between cycloplegic and non-cycloplegic refraction among 7–16 years-old children was observed in our previous study (33), which can reduce the errors in the estimation of myopia and estimated prevalence of myopia in this study. Furthermore, the procedure of non-cycloplegic autorefraction was time saving without the side effects of cycloplegia (e.g., photophobia, blurred near vision), especially in refraction screening of schools. Thirdly, two kinds of auto-refraction were used to measure the refraction of children, which may lead to measurement errors between different machines. Previous studies had found that the measurements of different kinds of auto-refraction had good agreement in young children (34–36). In addition, data on the environmental and genetic factors (e.g., outdoor activity time, near work time, parental income and myopia) were not collected in this study. Future study should be conducted to investigate the relationship between these factors and myopia in Shanghai and Yunnan.

## 5 Conclusion

Spherical equivalent and estimated prevalence of myopia of school-age children in Yunnan are similar to Shanghai. Furthermore, a similar situation exists between Han ethnicity and ethnic minorities in Yunnan. This similarity may be mainly due to the minimal differences in lifestyles and environmental exposures between Shanghai and Yunnan, and between Han ethnicity and ethnic minorities in Yunnan. Attention should also be paid to the eye health and prevention of myopia for children in rural areas and ethnic minorities. Longitudinal prospective studies with large sample size are required to provide further insight into the differences in myopia between urban and rural areas and among different ethnic population.

## Data Availability

The raw data supporting the conclusions of this article will be made available by the authors, without undue reservation.

## References

[1] Baird P Saw S Lanca C Guggenheim J Smith Iii E Zhou X Myopia. *Nat Rev Dis Primers*. (2020) 6:99. 10.1038/s41572-020-00231-4 33328468

[2] Morgan I French A Ashby R Guo X Ding X He M The epidemics of myopia: Aetiology and prevention. *Prog Retin Eye Res*. (2018) 62:134–49. 10.1016/j.preteyeres.2017.09.004 28951126

[3] Holden B Fricke T Wilson D Jong M Naidoo K Sankaridurg P Global prevalence of myopia and high myopia and temporal trends from 2000 through 2050. *Ophthalmology*. (2016) 123:1036–42. 10.1016/j.ophtha.2016.01.006 26875007

[4] Li T Jiang B Zhou X. Age-related change of axial length, spherical equivalent, and prevalence of myopia and high myopia in school-age children in Shanghai: 2014-2018. *J Ophthalmol.* (2020) 2020:4235893. 10.1155/2020/4235893

[5] Pan C Zheng Y Anuar A Chew M Gazzard G Aung T Prevalence of refractive errors in a multiethnic Asian population: The Singapore epidemiology of eye disease study. *Invest Ophthalmol Vis Sci*. (2013) 54:2590–8. 10.1167/iovs.13-11725 23513059

[6] Pan C Klein B Cotch M Shrager S Klein R Folsom A Racial variations in the prevalence of refractive errors in the United States: The multi-ethnic study of atherosclerosis. *Am J Ophthalmol.* (2013) 155:1129–38.e1. 10.1016/j.ajo.2013.01.009 23453694 PMC3759975

[7] Ma Y Wen Y Zhong H Lin S Liang L Yang Y Healthcare utilization and economic burden of myopia in urban China: A nationwide cost-of-illness study. *J Glob Health*. (2022) 12:11003. 10.7189/jogh.12.11003 35356656 PMC8934110

[8] Cheng C Wang N Wong T Congdon N He M Wang Y Prevalence and causes of vision loss in East Asia in 2015: Magnitude, temporal trends and projections. *Br J Ophthalmol*. (2020) 104:616–22. 10.1136/bjophthalmol-2018-313308 31462416

[9] Shi Y Ma D Li X Ethnic disparities in risk factors for myopia among han and minority schoolchildren in shawan, xinjiang, china. *Optom Vis Sci*. 2023 100:82–90. 36705718 10.1097/OPX.0000000000001949

[10] Sun J Zhou J Zhao P Lian J Zhu H Zhou Y High prevalence of myopia and high myopia in 5060 Chinese university students in Shanghai. *Invest Ophthalmol Vis Sci*. (2012) 53:7504–9. 10.1167/iovs.11-8343 23060137

[11] Ma Y Zou H Lin S Xu X Zhao R Lu L Cohort study with 4-year follow-up of myopia and refractive parameters in primary schoolchildren in Baoshan District, Shanghai. *Clin Exp Ophthalmol*. (2018) 46:861–72. 10.1111/ceo.13195 29577563 PMC6282580

[12] He X Sankaridurg P Xiong S Li W Naduvilath T Lin S Prevalence of myopia and high myopia, and the association with education: Shanghai Child and Adolescent Large-scale Eye Study (SCALE): A cross-sectional study. *BMJ Open*. (2021) 11:e048450. 10.1136/bmjopen-2020-048450 34949607 PMC8710858

[13] You Q Wu L Duan J Luo Y Liu L Li X Prevalence of myopia in school children in greater Beijing: The Beijing Childhood Eye Study. *Acta Ophthalmol*. (2014) 92:e398–406. 10.1111/aos.12299 25165786

[14] Wu L You Q Duan J Luo Y Liu L Li X Prevalence and associated factors of myopia in high-school students in Beijing. *PLoS One*. (2015) 10:e0120764. 10.1371/journal.pone.0120764 25803875 PMC4372519

[15] Wang S Guo Y Liao C Chen Y Su G Zhang G Incidence of and factors associated with myopia and high myopia in chinese children, based on refraction without cycloplegia. *JAMA Ophthalmol*. (2018) 136:1017–24. 10.1001/jamaophthalmol.2018.2658 29978185 PMC6142978

[16] Guo L Yang J Mai J Du X Guo Y Li P Prevalence and associated factors of myopia among primary and middle school-aged students: A school-based study in Guangzhou. *Eye*. (2016) 30:796–804. 10.1038/eye.2016.39 26965016 PMC4906452

[17] Pan C Chen Q Sheng X Li J Niu Z Zhou H Ethnic variations in myopia and ocular biometry among adults in a rural community in China: The Yunnan minority eye studies. *Invest Ophthalmol Vis Sci*. (2015) 56:3235–41. 10.1167/iovs.14-16357 26024108

[18] Wang M Cui J Shan G Peng X Pan L Yan Z Prevalence and risk factors of refractive error: A cross-sectional Study in Han and Yi adults in Yunnan, China. *BMC Ophthalmol*. (2019) 19:33. 10.1186/s12886-019-1042-0 30683073 PMC6347814

[19] Qian D Zhong H Li J Niu Z Yuan Y Pan C. Myopia among school students in rural China (Yunnan). *Ophthalmic Physiol Opt*. (2016) 36:381–7. 10.1111/opo.12287 26896871

[20] Jing S Yi X Lei Y Hu L Cheng W Wen T Prevalence and risk factors for myopia and high myopia: A cross-sectional study among Han and Uyghur students in Xinjiang, China. *Ophthalmic Physiol Opt*. (2022) 42:28–35. 10.1111/opo.12907 34704612

[21] Chin M Siong K Chan K Do C Chan H Cheong A. Prevalence of visual impairment and refractive errors among different ethnic groups in schoolchildren in Turpan, China. *Ophthalmic Physiol Opt*. (2015) 35:263–70. 10.1111/opo.12193 25783952

[22] Li T Wan T Yao X Qi H Chen X She M Time trend of axial length and associated factors in 4- and 5-year-old children in Shanghai from 2013 to 2019. *Int Ophthalmol*. (2021) 41:835–43. 10.1007/s10792-020-01637-5 33184676 PMC7943426

[23] Li T Zhou X Chen X Qi H Gao Q. Refractive error in Chinese preschool children: The Shanghai study. *Eye Contact Lens*. (2019) 45:182–7. 10.1097/ICL.0000000000000555 30260815 PMC6494031

[24] Li T Jiang B Zhou X. Axial length elongation in primary school-age children: A 3-year cohort study in Shanghai. *BMJ Open*. (2019) 9:e029896. 10.1136/bmjopen-2019-029896 31676647 PMC6830838

[25] Liao C Ding X Han X Jiang Y Zhang J Scheetz J Role of parental refractive status in myopia progression: 12-year annual observation from the Guangzhou twin eye study. *Invest Ophthalmol Vis Sci*. (2019) 60:3499–506. 10.1167/iovs.19-27164 31408112

[26] Xiong S Zhang B Hong Y He X Zhu J Zou H The associations of lens power with age and axial length in healthy Chinese children and adolescents aged 6 to 18 years. *Invest Ophthalmol Vis Sci*. (2017) 58:5849–55. 10.1167/iovs.17-22639 29141080

[27] Pan C Wu R Li J Zhong H. Low prevalence of myopia among school children in rural China. *BMC Ophthalmol*. (2018) 18:140. 10.1186/s12886-018-0808-0 29890943 PMC5996540

[28] Ding X Morgan I Hu Y Yuan Z He M. Exposure to the life of a school child rather than age determines myopic shifts in refraction in school children. *Invest Ophthalmol Vis Sci*. (2022) 63:15. 10.1167/iovs.63.3.15 35289844 PMC8934557

[29] Zhang Y Qiu K Zhang Q. Ametropia prevalence of primary school students in Chinese multi-ethnic regions. *Strabismus*. (2020) 28:13–6. 10.1080/09273972.2019.1665691 31544569

[30] Hu M Zhou Y Huang S Congdon N Jin L Wang X Population prevalence of myopia, glasses wear and free glasses acceptance among minority versus Han schoolchildren in China. *PLoS One*. (2019) 14:e0215660. 10.1371/journal.pone.0215660 30998750 PMC6472783

[31] Morgan I Wu P Ostrin L Tideman J Yam J Lan W IMI risk factors for myopia. *Invest Ophthalmol Vis Sci*. (2021) 62:3. 10.1167/iovs.62.5.3 33909035 PMC8083079

[32] Tedja M Wojciechowski R Hysi P Eriksson N Furlotte N Verhoeven V Genome-wide association meta-analysis highlights light-induced signaling as a driver for refractive error. *Nat Genet*. (2018) 50:834–48. 10.1038/s41588-018-0127-7 29808027 PMC5980758

[33] Li T Zhou X Zhu J Tang X Gu X. Effect of cycloplegia on the measurement of refractive error in Chinese children. *Clin Exp Optom*. (2019) 102:160–5. 10.1111/cxo.12829 30136309 PMC6585953

[34] Choong Y Chen A Goh PP. A comparison of autorefraction and subjective refraction with and without cycloplegia in primary school children. *Am J Ophthalmol*. (2006) 142:68–74. 10.1016/j.ajo.2006.01.084 16815252

[35] Wang D Jin N Pei R Zhao L Du B Liu G Comparison between two autorefractor performances in large scale vision screening in Chinese school age children. *Int J Ophthalmol*. (2020) 13:1660–6. 10.18240/ijo.2020.10.22 33078119 PMC7511371

[36] Wilson L Melia M Kraker R VanderVeen D Hutchinson A Pineles S Accuracy of autorefraction in children: A report by the American Academy of Ophthalmology. *Ophthalmology.* (2020) 127:1259–67. 10.1016/j.ophtha.2020.03.004 32317177

